# Open Fractures in National Football League Athletes: Analyzing Performance and Return to Sport

**DOI:** 10.51894/001c.87846

**Published:** 2023-12-05

**Authors:** Brandon Nudelman, Brandon B Gardner, Stewart A Bryant, Drew A Lansdown, Brian T Feeley, Nirav K Pandya

**Affiliations:** 1 San Francisco Orthopaedic Residency Program; 2 Southern California Orthopedic Institute; 3 Orthopaedic Surgery Advanced Orthopaedics and Sports Medicine; 4 Orthopaedic Surgery University of California San Francisco

**Keywords:** open fracture, National Football League, return to sport, performance

## Abstract

**INTRODUCTION:**

Open fractures are potentially devastating injuries for the professional athlete. We sought to compare return to sports (RTS) and performance in National Football League (NFL) athletes sustaining open versus closed fractures.

**METHODS:**

NFL athletes with surgically treated open and closed fractures of the forearm, tibial shaft, and ankle from 2009-2018 were identified through publicly available reports and records. Data including demographics, RTS, career duration, and the approximate value performance metric before and after injury were collected. Statistical analyses were performed comparing open to closed injuries. Continuous variables were compared using Mann-Whitney U or two sample t- tests while categorical variables were compared using Fisher’s exact test.

**RESULTS:**

Ninety-five athletes met inclusion criteria (10 open and 85 closed fractures). Overall, 90% (n = 9) returned to sport after an open injury and 83.5% (n = 71) returned after closed injury with a median time missed of 48.9 (range 35.1 – 117.4) weeks and 43.0 (range 2.4 – 108.0) weeks, respectively. Athletes undergoing forearm surgery were able to return sooner, at around 20.8 weeks, and ankle fractures conferred the lowest return rate at 80% (n = 48). There were no significant differences in career duration and post-injury performance between open or closed fracture cohorts.

**CONCLUSIONS:**

Although open fractures are relatively uncommon injuries seen in NFL athletes, our study suggests RTS for these players is high. Athletes undergoing surgical treatment for open fractures had similar RTS rates, performance metrics, and career durations compared to those with comparable closed fractures. This information can provide guidance for providers counseling elite athletes on postoperative expectations.

## INTRODUCTION

National Football League (NFL) athletes have the highest injury rate compared to professionals in other sports.^1^ Several studies exist investigating return to sport (RTS) and performance in professional athletes sustaining various injuries.^2,3^ Open fractures pose unique treatment challenges for the orthopaedic surgeon, especially mitigation of postoperative complications like nonunion or infection. These factors continue to be a source of significant morbidity relative to closed injuries.^4^ Depending on severity, risk of infection can range from 2%-50%.^5^ Open fractures tend to also raise concern whether an athlete will be able to RTS, let alone at the same level of performance.

Clinical outcomes are often measured in orthopaedics through validated scoring systems. However, post-surgical outcomes in professional athletes may be better measured through factors such as duration of career and on-field performance.^6,7^ With a better understanding of how open fractures have historically affected professional athletes, physicians can more accurately provide evidence-based counseling to the athlete regarding career expectations.

The purpose of this study was to: (1) determine RTS rates in NFL players following surgical treatment of open forearm, tibial shaft, and ankle fractures; (2) compare RTS rates, time missed, performance, and professional experience between open and closed injury cohorts; and (3) evaluate differences in RTS rates, time missed, performance, and professional experience based on fracture anatomic location.

## METHODS

Official injury reports for all 32 NFL teams from 2009-2018 seasons were reviewed, allowing for a 2-year minimum follow-up period. A comprehensive injury dataset prior to 2009 was not available to the public. Like previously published methodology,^3^ multiple sources of public information were scrutinized for evidence of athletes sustaining open and closed injuries. Archived records included player profiles, newspaper archives, press releases, and team injury reports. The initial search was conducted by two authors (BG and SB) and validated by a third author (BN). We chose to specifically evaluate forearm, tibial shaft, and ankle injuries, given the higher proportion of fractures in these regions compared to others such as humerus or femur. Institutional review board approval was not required for this research, as all information was publicly available.

### Inclusion Criteria

Two independent sources with reports consistent for surgical treatment of forearm, tibial shaft, and ankle fractures were required for inclusion, in addition to a minimum 10 NFL games played prior to injury. This criterion ensured an established presence in the league with adequate performance metrics for comparison.

Exclusion criteria included injury prior to being on an active professional team roster or during the rookie season, re-fractures, stress fractures, chronically treated osseous injuries, surgical treatment with suspensory fixation or arthroscopy alone, and inconclusive or insufficient evidence based on accessible reports.

Fracture type was classified based on anatomic location. Demographic variables including age, BMI (body mass index), and time away from sport were collected as well as information regarding professional experience and performance metrics through the 2020-2021 season. Players were categorized into the following positions: quarterback (QB), running back (RB), wide receiver (WR), tight end (TE), offensive lineman (OL), defensive lineman (DL), linebacker (LB), and defensive back (DB).

### Return to Sport and Career Duration

Dates of injury and RTS were collected. RTS was defined as participation in at least one regular season game following injury. Career duration was the number of years from first to final season on an NFL roster, or 2020 season if in active status, regardless of any seasons missed in full due to injury. Professional experience prior to injury included each season in which at least one regular season game was played, including that of the shortened injury year. Each season played in after return from injury was counted if the athlete played in a minimum of one game.

### Performance-Based Metrics

Performance-based metrics have been developed in attempt to quantify an athlete’s seasonal value. Many studies have used a more cumbersome model based on a previously published scoring system.^8,9^ We chose approximate value (AV) to help assess and compare an NFL athlete’s performance before and after injury. AV is a *Pro-Football-Reference* calculated seasonal value for an NFL player independent of player position or year. Data were collected on the www.pro-football-reference.com online database where detailed methodology of AV can be found. AV was identified in the two years preceding the injury shortened season, the two seasons immediately after injury and the maximum values in any season before and after injury. If the player was injured during a regular season game or offseason (i.e. 2015), then prior season’s AV (i.e. 2014) was selected for the first pre-injury data point. However, if injured during a playoff game or after game 14, then the data point from the same year’s regular season (i.e. 2015) was chosen. The first return season data point was chosen for the subsequent season (i.e. 2016), regardless of a midseason return or number or games played.

### Statistical Analysis

Statistical analysis was performed by author BG using Stata software version 16.1 (StataCorp). A Fisher’s exact test was used to analyze categorical data. Continuous variables were reported as the mean and standard deviation or median and range with comparisons performed using either a Mann-Whitney U test or two sample *t*-test, when appropriate. The Spearman correlation test explored associations of demographic, injury, and performance variables with RTS. A P-value < 0.05 was considered statistically significant.

## RESULTS

**Figure 1.**
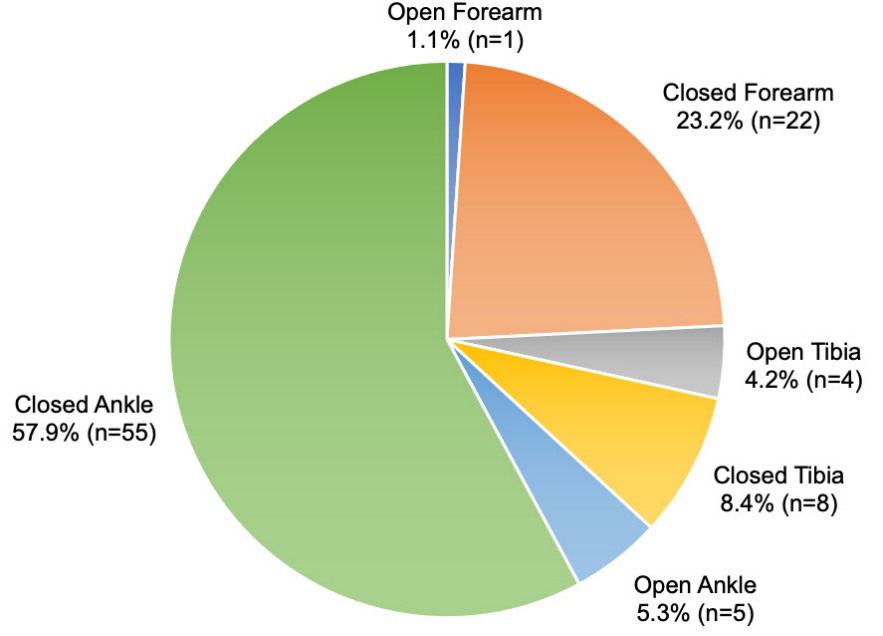
Distribution of open and closed fractures by anatomic location.

Ninety-five players met inclusion criteria of surgical treatment for injury after playing a minimum of 10 games. There were 10 (10.5%) surgically treated open injuries (1 forearm, 4 tibias, and 5 ankles) and 85 (89.5%) surgically treated closed injuries (22 forearms, 8 tibias, and 55 ankles) (P = 0.03, Figure 1, Table 1). There were three (30.0%) athletes identified with injury-related post-surgical complications/reoperations in the open cohort (2 infections, 1 re-fracture) compared to 13 (15.3%) in the closed cohort (3 infections, 5 re-fractures, 5 revision surgeries/hardware removal) (P = 0.36).

**Table 1.** Demographic Data and Injury Breakdown by Pattern and Player Position

Metric	Forearm		Tibia		Ankle		Entire Cohort		
	Open	Closed	Open	Closed	Open	Closed	Open	Closed	P value^c^
Injury type, n (%)^a^	1 (1.1)	22 (23.2)	4 (4.2)	8 (8.4)	5 (5.3)	55 (57.9)	10 (10.5)	85 (89.5)	0.03
QB		2	1	1		3	1	6	7 (7.4%)
RB		3	1	2	1	5	2	10	12 (12.6%)
WR		3	2	2	1	5	3	10	13 (13.7%)
TE		1			1	5	1	6	7 (7.4%)
OL						19		19	19 (20.0%)
DL					2	6	2	6	8 (8.4%)
LB		3		1		3		8	7 (7.4%)
DB	1	10		2		9	1	21	22 (23.2%)
Age at injury (years)^b^	24.0	29.2 ± 3.6	27.0 ± 4.7	26.9 ± 3.0	27.0 ± 1.2	26.3 ± 2.9	26.7 ± 3.0	27.1 ± 3.4	0.71
BMI (kg/m^2^)^b^	24.8	27.8 ± 2.2	26.6 ± 2.0	30.0 ± 4.4	31.9 ± 6.3	32.5 ± 5.3	29.1 ± 5.3	31.1 ± 5.0	0.24

QB=quarterback; RB=running back; WR=wide receiver; OL=offensive lineman; DL=defensive lineman; LB=linebacker; DB=defensive back; BMI=body mass index

<sup>a</sup>P value derived from two-sided Fisher’s exact test

<sup>b</sup>Reported as mean ± standard deviation (when n&gt;1, otherwise reported as absolute value); p-value derived from two sample <em>t</em>-test

<sup>c</sup>Total number of injuries by position denoted when P value not calculated</td>

### Injuries by Player Position

DBs (n = 22) followed by OL (n = 19) comprised the largest proportion of injuries (Table 1). The only positions to not sustain an open injury were OL and LBs. Of 23 forearm injuries, 11 (47.8%) occurred in DBs followed by 3 (13.0%) each for RBs, WRs, and LBs. For ankle injuries, 19 (31.7%) were in OL with DBs the next most represented at 9 (15.0%). Offensive players were more commonly injured, making up 7 (70.0%) and 51 (60.0%) of open and closed injury patterns, respectively. Fisher’s exact analysis comparing injury type by player position showed no significant difference between open and closed fractures (P = 0.14).

### Return to Sport (RTS)

Overall, 9 (90.0%) open fractures returned to sport and 71 (83.5%) surgically treated closed fractures returned to sport (P > 0.99, Table 2). RTS based on injury type (P = 0.34) and player position (P = 0.62) did not reveal any significant differences. Fifteen athletes did not return following surgical treatment for injury (Table 2). One athlete (20.0%) among those with an open ankle fracture (n=5) did not return. Among the closed injury cohort, two (9.1%) players with forearm fractures (n=22), one (12.5%) with a tibia fracture (n=8), and 11 (20.0%) with ankle fractures (n=55) were unable to RTS.

**Table 2.** Return to Sport Based on Injury Pattern and Position and Time to Return

Metric	Forearm		Tibia		Ankle		Entire Cohort		
	Open	Closed	Open	Closed	Open	Closed	Open	Closed	P value^c^
RTS, n (%)^a^	1 (100)	20 (90.9)	4 (100)	7 (87.5)	4 (80)	44 (80)	9 (90)	71 (83.5)	> 0.99
Unable to RTS, n (%)	0	2 (13.3)	0	1 (6.7)	1 (6.7)	11 (73.3)	1 (6.7)	14 (93.3)	15 (100%)
QB		1	0	0		0	0	1	1 (6.7%)
RB		0	0	1	0	1	0	2	2 (13.3%)
WR		1	0	0	0	1	0	2	2 (13.3%)
TE		0			0	1	0	1	1 (6.7%)
OL						4		4	4 (26.7%)
DL					1	2	1	2	3 (20.0%)
LB		0		0		1		1	1 (6.7%)
DB	0	0		0		1	0	1	1 (6.7%)
Time missed (weeks) ^b^	43.0	19.7 ± 17.9; 10 (2.4 – 49.6)	78.4 ± 36.5; 79.5 (37.1 – 117.4)	44.8 ± 6.7; 44.6 (36.0 – 54.3)	44.8 ± 6.6; 47.5 (35.1 – 49.0)	49.3 ± 17.3; 48.0 (5.0 - 108.0)	59.5 ± 28.9; 48.9 (35.1 – 117.4)	40.5 ± 21.2; 43.0 (2.4 – 108.0)	0.17

RTS=return to sport; QB=quarterback; RB=running back; WR=wide receiver; OL=offensive lineman; DL=defensive lineman; LB=linebacker; DB=defensive back

<sup>a</sup>P value derived from two-sided Fisher's exact test 
    <sup>b</sup>Reported as mean ± standard deviation and median with range (when n&gt;1, otherwise reported as absolute value); p-value derived from Mann-Whitney U test 
    <sup>c</sup>Total number of athletes by position unable to return to sport denoted when P value not calculated

The single athlete with an open forearm fracture returned postoperatively at 43.0 weeks, whereas 22 athletes with closed forearm fractures returned after a median 10.0 (range 2.4 – 49.6) weeks (Table 2). Of note, with one athlete in the open forearm cohort, we can only list an absolute return time value, and his return may have been delayed due to a concurrent ACL injury. Return from surgery after tibia fracture was 79.5 (range 37.1 – 117.4) and 44.6 (range 36.0 – 54.3) weeks for open versus closed injuries, respectively. Within the open tibia cohort, one athlete missed an additional year due to a tibia re-fracture in the preseason and another athlete missed an extra year due to an unrelated shoulder injury sustained during a preseason game. It took athletes 47.5 (range 35.1 – 49.0) and 48.0 (range 5.0 – 108.0) weeks to return after ankle fracture for open versus closed injuries, respectively.

Two of the nine (22.2%) returning athletes with open fractures missed the entire post-injury season compared to seven of the 71 (9.9%) returning athletes with closed fractures. 38.9% of athletes remained active by the end of the 2020-2021 season (n = 4 open, 40%; n = 33 closed, 38.8%). Injury type, player position, age, BMI, seasons played prior to injury, and pre-injury AV were not found to significantly correlate with RTS on Spearman’s correlation test.

The average career duration was 7.7 ± 3.2 and 8.0 ± 3.3 years for the open versus closed cohorts, respectively (P = 0.78, Table 3). The average age and professional experience at time of injury was 26.7 ± 3.0 years and 4.9 ± 3.4 seasons for open injuries and 27.1 ± 3.4 years and 5.0 ± 3.3 seasons for closed injuries (age: P = 0.71, Table 1; professional experience: P = 0.93, Table 3). Athletes able to RTS played on average another 2.4 ± 1.4 and 2.9 ± 2.0 seasons after sustaining open versus closed injuries, respectively (P = 0.60).

**Table 3.** Professional Experience Based on Return to Sport

Metric^a^	Forearm		Tibia		Ankle		Entire Cohort		
	Open	Closed	Open	Closed	Open	Closed	Open	Closed	P value
Professional experience pre-injury (n seasons)									
Non-returners (n=15)	-	9.0 ± 7.1	-	3.0	8.0	3.2 ± 2.6	8.0 ± 0	4.0 ± 3.7	-
Returners (n=80)	3.0	6.9 ± 3.5	5.0 ± 5.4	5.1 ± 3.3	4.5 ± 1.3	4.4 ± 2.7	4.6 ± 3.4	5.2 ± 3.2	0.55
All (n=95)	3.0	7.1 ± 3.8	5.0 ± 5.4	4.9 ± 3.1	5.2 ± 1.9	4.2 ± 2.7	4.9 ± 3.4	5.0 ± 3.3	0.93
Professional experience post-injury (n seasons)									
Returners (n=80)	3.0	2.8 ± 2.7	2.3 ± 1.5	3.6 ± 2.0	2.5 ± 1.7	2.8 ± 1.7	2.4 ± 1.4	2.9 ± 2.0	0.60
Career duration (years)									
Non-returners (n=15)	-	10.0 ± 5.7	-	4.0	8.0	4.5 ± 2.4	8.0 ± 0	5.3 ± 3.3	-
Returners (n=80)	6.0	10.0 ± 3.4	8.3 ± 5.3	9.1 ± 3.8	7.5 ± 1.3	7.8 ± 2.6	7.7 ± 3.4	8.6 ± 3.1	0.43
All (n=95)	6.0	10.0 ± 3.5	8.3 ± 5.3	8.5 ± 3.9	7.6 ± 1.1	7.2 ± 2.9	7.7 ± 3.2	8.0 ± 3.3	0.78

<sup>a</sup>Reported as mean ± standard deviation (when n>1, otherwise reported as absolute value) p-value derived from two sample <em>t</em>-test

### Performance Outcomes

For athletes returning to play, AV was collected and averaged for the two seasons preceding injury, which served as a baseline measure of performance at the time of injury. As compared to this pre-injury baseline, the first post-injury season AV decreased by 0.6 ± 2.4 points in the open injury cohort compared to a decrease of 0.4 ± 3.6 points in the closed injury cohort (P = 0.91). The two season post-injury average AV decreased by 2.1 ± 4.8 and 0.5 ± 3.2 points for open versus closed injury cohorts, respectively, as compared to the pre-injury two-year average baseline (P = 0.60, Table 4).

**Table 4.** Pre- and Post-Injury Performance Comparison for Athletes Returning to Sport

Metric	Forearm		Tibia		Ankle		Entire Cohort		
	Open	Closed	Open	Closed	Open	Closed	Open	Closed	P value
AV change post-injury vs pre-injury baseline^a,b^									
Post-injury season	1.0	-1.5 ± 2.7	-2.8 ± 4.2	0.3 ± 4.9	0.0 ± 0.6	-0.1 ± 3.9	-0.6 ± 2.4	0.4 ± 3.6	0.91
Post-injury 2- year avg	2.0	-1.4 ± 2.8	-4.6 ± 3.9	-0.6 ± 3.7	-0.5 ± 0.4	-0.2 ± 3.2	-2.1 ± 4.8	-0.5 ± 3.2	0.60
Post-injury max AV met or exceeded^c^									
Pre-injury 2- year avg, n (%)	1 (100)	12 (60)	2 (50)	3 (42.9)	2 (50)	28 (63.6)	5 (55.6)	43 (60.6)	> 0.99
Pre-injury max, n (%)	1 (100)	10 (50)	2 (50)	3 (42.9)	1 (25)	22 (50)	4 (44)	35 (49.3)	> 0.99

AV=approximate value

<sup>a</sup>Numerical data reported as mean ± standard deviation (when n&gt;1, otherwise reported as absolute value) p-value derived from Mann-Whitney U test 
    <sup>b</sup>pre-injury baseline calculated as the mean AV in the two seasons preceding injury< 
    <sup>c</sup>P value derived from two-sided Fisher's exact test

We also compared the maximum AV recorded by a returning athlete in any season after sustaining their surgically treated injury (Table 4). This value was compared to the pre-injury two-year baseline and the maximum AV recorded in any season prior to the studied injury. Five athletes (55.6%) with open injuries had an AV maximum that reached or exceeded the pre-injury baseline compared to 43 athletes (60.6%) with closed injuries (P > 0.99). Four (44.4%) and 35 (49.3%) reached or exceeded their pre-injury maximum AV (P > 0.99).

## DISCUSSION

Open fractures are potentially devastating, yet relatively uncommon injuries seen in professional athletes. For a professional athlete, successful treatment of an open fracture is defined by RTS, their on-field performance, and lack of complications.^3,10^ Thus, it is important to have some objective evidence regarding how high-level athletes fare after sustaining an open injury.

Our dataset includes ninety-five injuries, including 10 (10.5%) open injuries and 85 (89.5%) closed injuries spanning a 10-year period. Within the closed group, ankle injuries (n = 55, 64.7%) were most common followed by forearm injuries (n = 22, 25.9%). The RTS rate was comparable between open and closed fractures, with 90% (n = 9) of athletes successfully returning to their original field position after surgery for an open fracture compared to an 83.5% (n = 71) return rate for closed fractures. Return from time of injury took a median of 48.9 (35.1 – 117.4) versus 43.0 (2.4 – 108.0) weeks for open and closed fractures, respectively. Although this difference was not statistically significant, the nearly six week differential benefiting the closed cohort would surely represent a clinically significant amount of time missed for an NFL athlete due to absence from competition and loss of pay. This difference may have been influenced by two outliers in the open fracture cohort—one athlete suffering a serious infectious complication and another with an unrelated shoulder injury during a preseason game resulting in an additional year missed. Excluding these outliers, the return time decreases to 46.0 (35.1 – 59.9) weeks, only exceeding the closed cohort by three weeks. However, prolonged infectious complications are a serious concern for open fractures and excluding these cases may minimize the potential detrimental implications of an open injury. Calculating the median RTS rather than the mean helps minimize the influence of these outlying cases in our study sample. Of the 15 athletes (15.8%) in our dataset that did not RTS, none were identified as having a post-surgical complication. The inability to RTS may be multifactorial in nature and the definitive reasoning in our cohort is unknown. Only one athlete (10.0%) with an open injury (an open ankle) was unable to return, likely due to non-sport related etiologies.

Overall, athletes with forearm and tibia fractures experienced approximately 91% return rates, whereas those with ankle fractures experienced slightly lower rates of 80%. This is in line with a study by Mai et al who found a return rate of 96.3% after forearm surgery, 90.9% after tibial nailing, and 78.6% after ankle surgery.^3^ Another study found a return rate of 91.7% after forearm surgery.^11^

When interpreting our findings for time between games played, one must consider the small sample size for each injury type. By combining forearm, tibia, and ankle cohorts, regardless of open or closed patterns, median time between games played was 10.0 weeks, 49.0 weeks, and 48.0 weeks, respectively. Mai et al reported an average recovery period of 33.3 weeks for forearm surgery,^3^ but this may be due to less aggressive protocols in this earlier study period from 2003-2013. More recently, with data collected through 2016, Sochacki et al found athletes missed on average 21.7 weeks after forearm surgery.^11^ Mai et al’s results for the tibia and ankle were 51.3 weeks and 50.0 weeks, respectively.^3^

Our findings support existing evidence^3,11^ that athletes with forearm fractures are able to return more quickly compared to those with lower extremity injuries. This makes sense considering the lower demands on the upper extremity in football and the lower impact on mobility. The timing of injury (i.e. early or late in the season) can also impact the amount of time missed. In a study by Werner et al, the authors were able to report time missed as the duration between injury and return to full participation, even if return was achieved during the offseason. Thus, their cohort of surgically treated ankle fractures missed an average of 17.7 weeks.^12^ Although this time is about 30 weeks sooner than our ankle cohort, we feel return based on regular season gameplay is more accurate given the increased demands compared to activity during the offseason, practice, or pre-season play.

DBs accounted for half of the athletes with forearm fractures, and they were most injured overall. This finding is in line with previously published studies.^3,11,13^ DBs are generally smaller compared to other positions, which may explain an increased susceptibility to traumatic injuries. OL made up the highest proportion of ankle injuries, which was also the case in the Werner study.^12^ This is presumably a result of being frequently rolled-up on while blocking—the act of having another player incidentally fall and impact the lineman below the knee.

We chose AV as the primary modality to evaluate NFL athlete performance in the short and long term. This metric is simpler than previously used metrics, which require calculations based on a player’s on-field statistics. Additionally, AV is easily obtained from a professionally maintained public database and it uniquely allows inter-positional comparisons. It has been the preferred performance metric for other authors including a study on Achilles tendon ruptures and another on shoulder instability.^10,14^ In these studies, AV was significantly decreased in the two years after sustaining an Achilles tendon rupture, but not significantly affected after a shoulder instability event. In our study, there were no statistically significant differences in performance values when comparing athletes with open and closed injuries in the immediate postoperative season. To better analyze long-term effects, we compared maximum performance values achieved before and after sustaining injury. Outperforming the pre-injury maximum suggests an athlete eventually played at a level that was at or better than before undergoing surgery. Since injuries tended to occur in the latter half of careers, some of the performance decline may be attributable to natural regression.

The NFL Players Association estimates that player careers average 3.8 years, but a player who makes an opening day roster can expect an average career exceeding six years.^15^ The athletes in our dataset, who were on active rosters, had an average career duration around eight years. Athletes in both open and closed cohorts essentially played the same number of seasons before and after injury (4.9 vs 5.0 before and 2.4 vs 2.0 after, respectively). Interestingly, injuries typically occurred in the second half of a playing career, suggesting an association with increasing tenure. The relatively short NFL career only heightens the importance to rehabilitate athletes to a professional level of play as quickly as possible.

Although open fractures do not commonly occur in NFL athletes, our findings are of particular interest for setting postoperative expectations. Players can be counseled that return to play rates and performance are similar to comparable closed injuries. Various factors, such as complications, may increase return time. While this may be the case for elite-level football players with unlimited support services and recovery resources, results may differ in lower-level athletes or cases with high-energy mechanisms.

This study is not without limitation, including the inherent limitations of a retrospective study design, potentially inducing bias and limiting strength of conclusions. The open fracture sample size was small, which in one group prevented calculating a mean/median, and likely contributed to large standard deviations. Also, this may have resulted in type II error, limiting our ability to detect significant differences and impacting the generalizability of our findings. Furthermore, we relied on public reporting to identify our study cohort, which may introduce selection or reporting bias. However, previous studies have successfully utilized public reporting in their methodology.^3,6,8–11,14^ We did attempt to minimize error by confirming injuries on at least two separate reports. Additionally, we utilized strict inclusion criteria and limited our capture to a recent decade, improving identification accuracy and reducing misclassification bias. Lastly, we were unable to classify fracture patterns or soft tissue severity, a similar challenge encountered by others utilizing public reports to investigate fractures in professional athletes. Authors with access to the NFL injury database could potentially study open fractures with a better understanding of the full injury profile. We recognize that the variability among fractures, unrelated injuries during an athlete’s career, and other factors are difficult to ascertain and may influence outcomes. Again, we attempted to account for this through strict inclusion criteria while carefully identifying acute forearm, tibial shaft, and ankle fractures undergoing the appropriate operative treatments.

## CONCLUSION

Open fractures of the forearm, tibia, and ankle are relatively uncommon injuries seen in NFL athletes. Our study suggests return to sport rates for these players is high, reaching 90% in our cohort. Players undergoing surgical treatment for open fractures had similar return to sport rates, performance metrics, and career durations compared to players with similar closed fractures. This information can provide guidance for providers counseling elite athletes on postoperative expectations. Eventually, re-evaluation with a larger sample size should be the target of future investigation.

### Conflict of Interest

None
